# Caesarean Delivery and Postpartum Maternal Mortality: A Population-Based Case Control Study in Brazil

**DOI:** 10.1371/journal.pone.0153396

**Published:** 2016-04-13

**Authors:** Ana Paula Esteves-Pereira, Catherine Deneux-Tharaux, Marcos Nakamura-Pereira, Monica Saucedo, Marie-Hélène Bouvier-Colle, Maria do Carmo Leal

**Affiliations:** 1 Inserm UMR 1153, Obstetrical, Perinatal and Pediatric Epidemiology Research Team, (Epopé), Center for Epidemiology and Statistics Sorbonne Paris Cité (CRESS), DHU Risks in pregnancy, Paris Descartes University, Paris, France; 2 Department of Epidemiology and Quantitative Methods in Health, Sérgio Arouca National School of Public Health, Oswaldo Cruz Foundation, Rio de Janeiro, RJ, Brazil; 3 National Institute of Women, Children and Adolescents Health Fernandes Figueira, Oswaldo Cruz Foundation, Rio de Janeiro, RJ, Brazil; London School of Economics, UNITED KINGDOM

## Abstract

**Background:**

Cesarean delivery rates continue to increase worldwide and reached 57% in Brazil in 2014. Although the safety of this surgery has improved in the last decades, this trend is a concern because it carries potential risks to women’s health and may be a modifiable risk factor of maternal mortality. This paper aims to investigate the risk of postpartum maternal death directly associated with cesarean delivery in comparison to vaginal delivery in Brazil.

**Methods:**

This was a population-based case—control study performed in eight Brazilian states. To control for indication bias, deaths due to antenatal morbidity were excluded. We included 73 cases of postpartum maternal deaths from 2009–2012. Controls were selected from the Birth in Brazil Study, a 2011 nationwide survey including 9,221 postpartum women. We examined the association of cesarean section and postpartum maternal death by multivariate logistic regression, adjusting for confounders.

**Results:**

After controlling for indication bias and confounders, the risk of postpartum maternal death was almost three-fold higher with cesarean than vaginal delivery (OR 2.87, 95% CI 1.63–5.06), mainly due to deaths from postpartum hemorrhage and complications of anesthesia.

**Conclusion:**

Cesarean delivery is an independent risk factor of postpartum maternal death. Clinicians and patients should consider this fact in balancing the benefits and risks of the procedure.

## Introduction

Caesarean section (CS) rates continue to increase worldwide, with variations amongst countries and regions [1, 2]. In Brazil, CS rates have rapidly increased in the last 30 years, reaching 57% in 2014 [3]. This increase is not likely due to an extreme change in obstetrical risk but rather an expansion of the range of the indications of CS. Indeed, 84% of CS deliveries in Brazil are performed before the onset of labour [4], most likely for non-medical reasons [5, 6]. Caesarean section has been associated with multiple risks to women’s health [7–11] and may be a modifiable risk factor of maternal mortality, but this remains controversial.

Maternal mortality ratio (MMR) is the number of women who die from pregnancy-related causes while pregnant or within 42 days of pregnancy termination per 100,000 live births. It is a marker of the performance of health services, because most maternal deaths are avoidable if all women have convenient access to good-quality care. In Brazil, the MMR remains at a high level (60·8/100000 live births in 2011) [12], with modest improvement in the last decade. Despite the importance of the issue, few studies in the country have contributed to the understanding of its determinants [13].

A review of the literature on the relationship between CS and maternal mortality, published in 2006, found inconsistent results and concluded that no study had an ideal design or adequate power to establish this relationship [14]. Subsequent studies were also limited because of insufficient power to study mortality or because they failed to consider the potential “indication bias”, whereby antenatal morbidity may be the indication for CS and the cause of maternal death [8, 10, 15]. Although different conceptual approaches have been proposed to address this indication bias—selection of cesarean deliveries for which this bias was unlikely [9]; selection of causes of death for which this bias was unlikely, based on their timing of occurrence [16, 17] or on the judgment of investigators [18]; and adjustment on preexisting conditions—to our knowledge, only three published studies have considered this indication bias. One lacked external validity in that it was restricted to one university hospital in India [16]; the other two were conducted in France [17] and Canada, [9] and whether their results are transposable to less developed countries is questionable. In the absence of randomized controlled trials to assess benefits and risks of CS, high quality observational studies to elucidate risks are very important. Brazil is an upper—middle-income country, with a high rate of CS and persisting high MMR, providing an exemplary and propitious context to analyse this relationship. The recent Birth in Brazil study offers the opportunity of a national representative sample of parturient women who can serve as a reference population [19, 20]. In this case—control study, we aimed to investigate the risk of postpartum maternal death associated with CS by comparison to vaginal delivery (VD), globally and by the main causes of death. The study involved eight Brazilian states with the highest coverage and quality of data from the Mortality Information System (Sistema de Informação sobre Mortalidade) (SIM) and Maternal Mortality Enquiry Committees (MMECs).

## Methods

### Selection of cases

In the last two decades, the Brazilian government has been creating and improving MMECs and establishing a number of initiatives to expand the coverage and enhance the quality of the SIM such as setting goals to increase the coverage of mortality data, strategies to reduce ill-defined causes of death and integration of the SIM with other information systems [21].

The MMECs use the World Health Organization (WHO) International Classification of Diseases definition of a maternal death: “the death of a woman while pregnant or within 42 days of termination of pregnancy, irrespective of the duration and site of the pregnancy, from any cause related to or aggravated by pregnancy or its management but not from accidental or incidental causes” [22]. Since 2003, maternal deaths are an event of compulsory notification, mandating the investigation of deaths of women of childbearing age (10–49 years old) whose causes can hide maternal death (presumptive). Deaths with any mention of pregnancy, birth, or puerperium on review of the death certificate’s content, as well as presumptive maternal deaths, must be reported to and investigated by the MMECs [22]. MMECs are centralized at the state level, and all states use the same standardized detailed abstraction form to collect relevant clinical information related to the woman and her death [22]. This information is collected from interviewing the family, from the medical records and from the death certificate. The death certificate in Brazil allows the assignment of the underlying cause of death and up to three subsequent and two contributing causes of death. Each maternal death is reviewed by the state committee of experts, which define the cause of death based on all the information collected.

For the current study, we first identified women who died within 42 days after delivery, from 2009–2012, in the eight states with the highest coverage and quality of data from SIM and MMECs: Espírito Santo, Rio de Janeiro, São Paulo, Paraná, Santa Catarina, Rio Grande do Sul, Mato Grosso do Sul and Distrito Federal. We performed a probabilistic linkage [23] between the maternal deaths and the Information System on Live Births (Sistema de Informação sobre Nascidos Vivos) (SINASC) [24], for live births and with SIM [25], for stillbirths. Then, to be consistent with the selection of the controls (see below), we restricted the study to women who died after having delivered in one of the hospitals sampled for the Birth in Brazil Study [19, 20] within these eight states. The four-year period to collect the cases was determined in order to include a number of cases allowing adequate sample size, and to be consistent with the period of inclusion of the controls; the pattern of causes of maternal deaths in Brazil did not change during this period [12].

Amongst those 274 postpartum maternal deaths, we excluded women with multiple pregnancies and women whose cause of death was from a condition present before the onset of labour likely to also affect their probability of having a CS (selection by indication bias) [17]. The deaths excluded were deaths due to chronic conditions present before pregnancy (circulatory-system, hematologic, digestive-system and respiratory-system diseases; mental disorders; neoplasm; and chronic infection); and deaths due to obstetric conditions that developed during pregnancy but before the onset of labour (hypertensive disorders in pregnancy, haemorrhage due to placenta praevia or accreta and abruptio placentae, amniotic fluid embolism, cerebral venous thrombosis, intracerebral haemorrhage and chorioamnionitis). The information available for our review for this classification was the data from the MMECs summary sheet, as well as the information from the death certificate, before and after amendments of the MMECs. We reviewed the whole content of the summary sheet and the death certificate to identify exactly the time when the complication began, and not only the time of death, to properly exclusion of deaths. When there was not enough information on the death certificate to identify exactly the time when the complication began, we reviewed the medical records from the hospital where the women delivered. That was necessary for 28 cases of maternal deaths. Amongst the remaining 80 deaths, we excluded deaths from deliveries in private hospitals because preliminary analyses showed an interaction between CS and the private status of the unit, and the small number of deaths in these hospitals (seven deaths) did not allow a stratified analysis in this subgroup.

**Fig 1:**
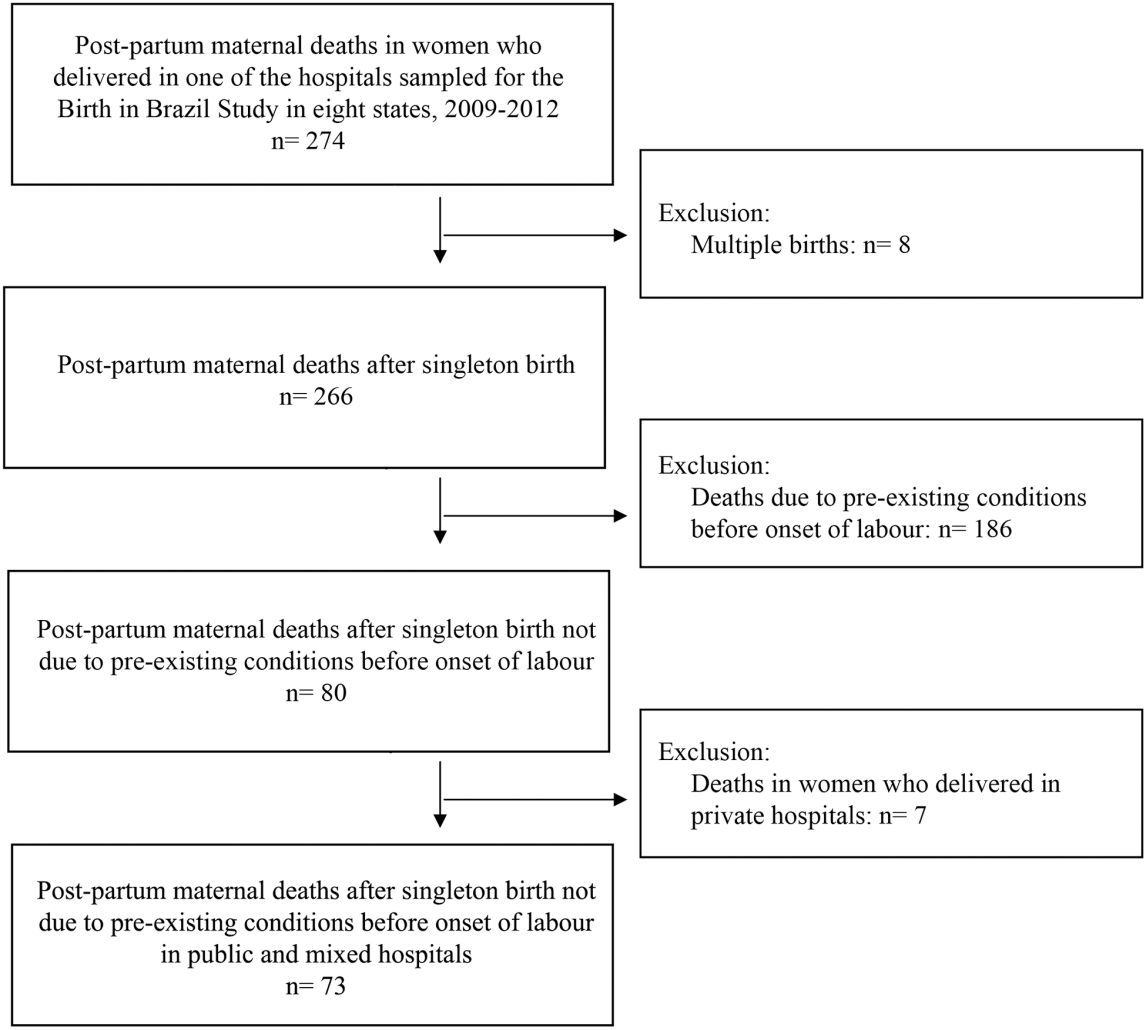
Selection of cases.

The remaining 73 cases were defined as women who delivered in public or mixed hospitals and died within 42 days postpartum after a pregnancy that resulted in a singleton birth, from causes not due to conditions or from complications present before the onset of labour. Fig 1 summarizes the process of defining cases.

### Selection of controls

The controls were selected from the Birth in Brazil Study, a nationally representative, hospital-based study of parturient women and their newborns performed in 2011. The Birth in Brazil Study had a complex sample of 266 hospitals and a total of 23,940 postpartum women interviewed throughout Brazil. The sample is described elsewhere [19]. All women who had given birth to a live newborn, regardless of weight or gestational age, or to a stillbirth with birth weight ≥ 500 g and/or gestational age ≥ 22 weeks of pregnancy in one of the sampled hospitals during the data collection were invited to participate in the Birth in Brazil Study. Face-to-face interviews were held with the postpartum women during their hospital stay to collect information on sociodemographic characteristics such as age, skin colour and years of schooling. Further clinical data about the women and their newborns were collected from their medical records and extracted from photographs of prenatal care cards. More details on the data collection have been published elsewhere [20].

For the present study, we selected Birth in Brazil participant women included in the eight states (94 public or mixed hospitals) where the cases were selected. We applied for the controls the same selection criteria as for the cases. We excluded women with multiple pregnancies (88 women), maternal deaths (two women) and maternal “near misses” based on the WHO criteria (103 women) (S1 Fig).

We conducted a sensitivity analysis with three different scenarios of exclusion criteria for the controls. One: only excluding multiple pregnancies (88 women) and maternal deaths (two women). Two: scenario one plus excluding maternal “near misses” based on the WHO criteria (103 women) [25, 26]—the one applied in this article—and three: scenario two plus excluding women with obstetric conditions potentially related to emergencies before the onset of labour (eclampsia, placenta praevia or accreta, placental abruption and severe infection) (45 women).

After applying the sample weights for cases and controls [19], this study included 73 cases and 9,221 controls.

### Variables

The primary predictor variable was type of delivery, caesarean section or vaginal. The covariates examined as potential confounders in the association between type of delivery and maternal mortality were mother’s age, mother’s years of education, parity, previous CS, premature delivery, macro-region and hospital of delivery (public or mixed). For cases, information on these variables was collected from the birth certificate provided electronically by SINASC [24]. Data from birth certificate has been shown to be very reliable in Brazil in the last decade [24, 27]. For controls, information was collected from medical records. We had complete data for all 73 cases (100.0%) and 9,073 controls (98.4%)—the missing values were for the variables “age” (two) “years of education” (23) “previous CS” (31) and premature delivery (115).

### Statistical analysis

Post-hoc calculations showed that with a significance level of 5%, this sample would have 80% power to detect an increased risk of death after CS as compared with VD for a corresponding OR of ≥ 2.0.

We analyzed the differences in women’s characteristics between cases and controls by χ^2^ test. The association between type of delivery and maternal death, globally and by causes, was examined by univariate and multivariate logistic regression. Crude and adjusted OR and 95% confidence intervals (CI) were calculated taking into account the sample weights and the complex sampling design. We adjusted for the covariates age (10–19, 20–24, 25–29, 20–34, ≥35), years of education (≤3, 4–7, 8–11, ≥12), parity (0, 1–2, ≥3), premature delivery (yes, no), region (South, Southeast, Midwest), type of hospital (public and mixed) and previous CS (0, 1, ≥2). We included the region and type of hospital in the models because the CS rate amongst controls varied by these variables. We conducted the same analyses, globally and by causes. A subgroups analysis was conducted with the term deliveries only (gestational age ≥37 weeks).

Interactions between type of delivery and the covariates region, type of hospital, age, schooling, parity and previous CS were tested. In all statistical analyses the complex sampling design was taken into consideration. The level of statistical significance was 0.05. SPSS 22.0 was used for analysis (IBM Corp., Armonk, USA).

### Details of ethics approval

This study was carried out in accordance with the National Health Council Resolution n. 196/96. The ethics committee of the Sérgio Arouca National School of Public Health, Oswaldo Cruz Foundation (CEP/ENSP), approved the Birth in Brazil study and the current case-control study under the research protocols CAAE: 0096.0.031.000–10 (approval date: May 11^th^ 2010) and CAAE: 32359614.9.0000.5240 (approval date: November 13^th^ 2014), respectively. All hospital directors and postpartum women controls subjects signed an informed consent form to participate in the Birth in Brazil Study. We personally obtained data about postpartum mortality case subjects from the Secretary of Health Surveillance (Secretaria de Vigilância em Saúde) of Ministry of Health, Brasília, DF, Brazil, which centralize at a national level the electronic registries from the States Maternal Mortality Enquiry Committees. Informed consent for the case subjects was obtained from the next of kin by the States Committees, thus the Secretary of Health Surveillance and the ethics committee of the Sérgio Arouca National School of Public Health waived the requirement for additional informed consent for case subjects.

## Results

This study included 73 cases and 9,221 controls. Cases were significantly older, had fewer years of education, and were more likely to have three or more previous deliveries, two or more previous CS and to have delivered preterm (Table 1). Amongst controls, the same variables, plus region and type of hospital, were associated with CS (S1 Table).

**Table 1:** Sociodemographic and birth characteristics amongst cases and controls.

	Cases		Controls		
	n	%	n^a^	%	P-value*
**All**	73	100	9,221	100.0	
**Region**					
** Southeast**	48	65.3	6,152	66.7	0.958
** South**	20	27.8	2,488	27.0	
** Midwest**	5	6.9	581	6.3	
**Type of hospital**					
** Public**	33	45.2	3,863	41.9	0.568
** Mixed**	40	54.8	5,358	58.1	
**Age in years**					
** 10–19**	15	20.5	1,760	19.1	0.008
** 20–24**	10	13.7	2,814	30.5	
** 25–29**	17	23.3	2,196	23.8	
** 30–34**	21	28.8	1,534	16.6	
** ≥ 35**	10	13.7	915	9.9	
**Years of education**					
** ≤ 3**	8	11.0	285	3.1	<0.001
** 4–7**	22	30.1	1,870	20.3	
** 8–11**	37	50.7	5,655	61.5	
** ≥ 12**	6	8.2	1,388	15.1	
**Skin color**					
** White**	38	52.1	5,085	55.2	0.594
** Non white**	35	47.9	4,132	44.8	
**Number of previous deliveries**					
** 0**	21	28.8	4,128	44.8	0.001
** 1 to 2**	35	47.9	4,136	44.9	
** ≥ 3**	17	23.3	957	10.4	
**Previous c-section**					
** Primiparous**	21	28.8	4,128	44.9	
** Multiparous**					
** 0**	30	41.1	2,969	32.3	0.003
** 1**	12	16.4	1,573	17.1	
** ≥ 2**	10	13.7	520	5.7	
**Premature delivery**					
** No**	52	71.2	8,185	89.9	<0.001
** Yes**	21	28.8	921	10.1	

^a^ For each covariate the sum of controls varied (9,106 to 9,221) according to the number of missing values.

* χ2 test.

The proportion of CS was 64.4% (47/73) for cases and 46.9% (4256/9073) for controls. After adjusting for potential confounders, CS was associated with a significantly increased risk of postpartum maternal mortality, adjusted OR 2.9 (95% CI 1.6–5.1) (Table 2). The analysis conducted with the term deliveries only provided comparable results (S2 Table).

**Table 2:** Postpartum maternal mortality associated with caesarean delivery.

	Cases (73)		Controls (9,073)		Crude		Adjusted	
	n	%	n	%	OR	95% CI	OR adj.*	95% CI
**Vaginal**	26	35.6	4,817	53.1	-	-	-	-
**Cesarean**	47	64.4	4,256	46.9	2.0	(1.3–3.3)	2.9	(1.6–5.1)

*Adjusted for region, type of hospital, age, schooling, parity, premature birth and previous caesarean delivery.

Amongst cases, the causes of postpartum maternal deaths differed between CS and VD (P = 0.03, χ^2^ test). Postpartum haemorrhage accounted for about half of the deaths for both types of delivery (Table 3).

**Table 3:** Causes of death by type of delivery amongst cases.

	Cases (73)			
	Vaginal		Caesarean	
Causes of death	n	%	n	%
**Global causes**	26	100.0	47	100.0
**Postpartum haemorrhage**	14	53.8	26	55.3
**Puerperal infection**	10	38.5	8	17.0
**Thromboembolism**^a^	2	7.7	5	10.6
**Amniotic fluid embolism**	0	0.0	1	2.1
**Complications of anaesthesia**^b^	0	0.0	5	10.6
**Unspecified obstetric death**^c^	0	0.0	2	4.3

^a^ Includes one case of cerebral venous thrombosis in the Caesarean group.

^b^ Four cases of general anaesthesia and one case of spinal anaesthesia.

^c^ Unspecified obstetric deaths after hospital discharge.

*P-value* of χ2 test = 0.03

Considering the cause-specific postpartum maternal mortality, CS was associated with a significantly increased risk of death from postpartum haemorrhage (adjusted OR 3.0; 95% CI 1.4–6.6) (Table 4) and accounted for all deaths from complications of anaesthesia (Table 3). The risk of death from thromboembolism associated with CS did not differ significantly from VD (OR 3.5; 95% CI 0.5–26.4), (Table 4).

**Table 4:** Cause-specific postpartum maternal mortality associated with caesarean delivery.

	Cases (73)				Controls (9,073)				Crude		Adjusted	
	Vaginal		Caesarean		Vaginal		Caesarean					
	n	%	n	%	n	%	n	%	OR	95% CI	OR adj.*	95% CI
**Global causes**	26	35.6	47	64.4	4,817	53.1	4,256	46.9	2.0	(1.3–3.3)	2.9	(1.6–5.1)
**Postpartum haemorrhage**	14	35.0	26	65.0					2.1	(1.1–4.0)	3.0	(1.4–6.6)
**Puerperal infection**	10	55.6	8	44.4					0.9	(0.4–2.3)	1.2	(0.4–3.5)
**Thomboembolism**^a^	2	28.6	5	71.4					2.3	(0.4–12.4)	3.5	(0.5–26.4)
**Thomboembolism**^a^ **and other causes combined**^b^	2	13.3	13	86.7					7.4	(1.7–32.6)	10.9	(2.2–55.3)

* Adjusted for region, type of hospital, age, schooling, parity, premature birth and previous caesarean delivery.

^a^ Includes one case of cerebral venous sinus thrombosis in the group of caesarean.

^b^ Eight cases in the caesarean group: one from amniotic fluid embolism, four from general anaesthesia, one from spinal aesthesia and two unspecified obstetric death after hospital discharge.

The risk for CS due to amniotic fluid embolism, complications of anaesthesia and unspecified obstetric death could not be estimated because there were no maternal deaths due to these causes after vaginal delivery. We grouped them with deaths from thromboembolism in order to estimate the risk for CS from causes other than postpartum haemorrhage and infection. CS was associated with increased risk of death from these causes combined (OR 10.9; 95% CI 2.2–55.3) (Table 4).

We did not find significant interactions between CS and the covariates region, type of hospital, age, schooling, parity and previous CS for risk of maternal mortality. Results did not differ significantly when other criteria for selecting the controls were used, i.e not excluding maternal near misses from controls or widening the exclusion to women with obstetric conditions potentially related to emergencies before delivery (S3 Table).

## Discussion

### Main Findings

After adjusting for potential confounders, the risk of postpartum maternal mortality was almost threefold higher with CS than VD. The increased risk was mainly due to deaths from postpartum haemorrhage and complications of anaesthesia.

### Strengths and Limitations

This study has several strengths. The Brazilian context can be considered as exemplary of middle- income countries with high CS rates. The Birth in Brazil Study is the first national enquiry into labour and birth in Brazil, so we could select the controls from a representative and reliable database [20]. Most previous Brazilian studies on maternal mortality were restricted to a locality (city or state), were descriptive or focused on methods to improve the estimation of the ratio [13]. Only recently did information from the SIM (enhanced by the MMECs) became more trustworthy [12], and in the present study, maternal deaths and their causes were assessed by these committees, based on detailed information on the circumstances of death. Finally, we accounted for “indication bias” and selected the cases after excluding all maternal deaths attributed to pre-existing morbidities. The main strategy was to exclude deaths due to conditions present before the onset of labour so as to estimate the risk solely from CS. If these deaths were not excluded, the same analysis would have led to an apparently much stronger association between CS and maternal mortality (proportion of CS amongst cases would have been 80%). We cannot completely exclude that some indication bias remains, as we did not take into account all ante partum condition that could have influenced the fatal issue, but only ante partum conditions that were the causes of deaths. However, such a bias is very unlikely to entirely explain the observed effect, given the presumed mildness of remaining ante partum conditions as well as the strength of the association found here (adj. OR of 3). For the controls, being conservative in the exclusion criteria and only excluding maternal deaths and nearmisses, may have slightly underestimated our OR. However, the sensitivity analysis testing various selections of controls showed that this did not modify our results (S3 Table).

One limitation of this investigation is the possible underreporting of some maternal deaths, although the Brazilian MMECs coverage is increasing each year. Using a specific methodology to measure the extent of the underreporting in Brazil, Szwarcwald et al (2014) found a national adjustment factor of 1.42 for the year 2011 [12]. To limit this potential underreporting, we analyzed the deaths in eight Brazilians states in which both the exhaustivity of investigation and quality of data are higher than the average for Brazil, thus increasing the internal validity of our estimates. Our results may not be generalizable to the overall population because we did not include private hospitals. However, our study covered most of the population of these states. Home births account for < 1% of births in Brazil, and public and mixed hospitals represent almost 80% of all deliveries [3]. In the current analysis we did not exclude assisted vaginal deliveries, which represent a high risk situation. However, it is unlikely that it influenced our results given the very low prevalence found in our control sample (1.5%). Moreover, not excluding them would actually lead to a conservative bias toward the null hypothesis—as it is expected to find more assisted vaginal deliveries among cases than among controls. Finally, we were not able to study the risk of postpartum death by the timing of the CS, prelabour or intrapartum. Neverthless, in Brazil, most CS are performed before the onset of labour (84%) [4] and our OR are reasonably representing the risk of this type of CS. It is important to mention that, although both types of CS were analysed together, cases with intrapartum complications such as abruption placentae or obstructed labour reported in any line of the death certificate were excluded, as well as deaths due to hemorrhage caused by conditions present before delivery—i.e placenta praevia ou accrete.

### Interpretation

Another study with an appropriate design to test the relationship between CS and postpartum maternal death was a Canadian retrospective cohort study comparing otherwise low-risk women who delivered by planned CS because of breech position in women with planned VD [9]. The authors found that planned CS deliveries were associated with increased risk of specific severe postpartum complications but not increased risk of maternal death, which was only higher for emergency CS [9]. However, the focus of the study was severe maternal morbidity and the number of women with elective CS was too limited to relevantly test the association with maternal mortality, given the very low ratio of maternal mortality in this country [9].

Our results agree with those from the WHO Global Survey on maternal and perinatal health in Latin America, finding elective CS independently associated with maternal mortality (OR 3.4) [10]. However, this study did not investigate the risk of death associated with CS by the main causes of death and did not account for indication bias [10]. Another WHO Global Survey on maternal and perinatal health from Asia accounted for indication bias by grouping the CS as with or without indication. Both antepartum and intrapartum CS performed without indication were associated with severe maternal outcome (maternal deaths and severe morbidity combined) with adjOR of 2.7 and 14.2, respectively. However, the number of deaths was too small for a statistically significant association between each type of CS and maternal deaths alone [28].

We found that CS was associated with maternal deaths from postpartum haemorrhage. In a WHO survey including 28 countries, intrapartum CS was one factor associated with postpartum haemorrhage diagnosis [29], and a recent study from Brazil reported that CS was one of the main factors associated with increased risk of severe maternal morbidity secondary to postpartum haemorrhage [30]. Regarding deaths, CS risks are likely to vary between settings not only because of the varying capacity between settings/hospitals/countries to properly conduct safe surgery, but also the capacity to treat surgical complications. This may be the reason our results contrast with findings from France [17], where CS was not associated with increased risk of death from postpartum haemorrhage. Although loss of blood is much greater in CS than VD deliveries, in France, women with CS likely receive more thorough postpartum surveillance and care than in Brazil, thus decreasing the delay of diagnosis and management of postpartum haemorrhage. Another difference may be in the availability of blood bank or transfusion services, which are lacking even in maternity wards with ≥500 births/year in Brazil (coverage of around 70%) [31]. Further studies on blood loss after CS are needed to assess the adequacy of care and to address both the availability of blood and transfusion services as well as other means to prevent and treat haemorrhage [32].

Our results indicate that CS is associated with increased risk of postpartum maternal deaths from complications of anaesthesia. All five deaths from this cause occurred after CS, four after general anaesthesia and one after spinal anaesthesia. In Brazil, <1% of CS cases require general anaesthesia (unpublished data from the Birth in Brazil Study). This finding agrees with previous knowledge that general anaesthesia represents greater risk in the context of pregnancy than does spinal anaesthesia and is an option considered with extreme criteria. However, general anaesthesia is likely performed in the most severe cases and so isolating its independent effect is difficult.

Our lack of finding CS associated with deaths from postpartum infection is surprising because this is one of the most frequent post-operative complications of any surgery. In France, CS was strongly associated with mortality from postpartum infection [17]. In Canada, the risk of major puerperal infection was higher after planned CS than planned VD [9], and in Latin America CS was associated with increased use of antibiotics [7]. Infection has been cited as one of the causes of rehospitalisation after CS [33]. The lack of association we found may have been due to a high contribution of sepsis to deaths after VD. Indeed, more than a third of maternal deaths after a VD in our group of cases were due to infection. A recent Brazilian network study found an association between delay in obstetric care and severity of adverse maternal outcomes, with infections associated with a high prevalence ratio for any delay as compared with other diagnoses [34,35]. This finding would explain the high proportion of maternal deaths due to infection (46,6%) despite the lower contribution of infection to cases of maternal morbidity (5.3%) [34,35]. Another possible explanation is that appropriate and timely treatment of infection can vary according to type of delivery in the Brazilian context, leading to a high lethality after VD—an aspect that should be investigated. Further analyses including both cases of severe maternal morbidity and mortality will broaden knowledge and discussion about the impact of CS on specific morbidities on one hand and fatal issues on the other hand.

Caesarean section is an important procedure in current obstetrics and is often used to resolve at-risk situations for both the mother and the fetus. The relative risk of death associated with CS was similar to that reported in high-resource countries [9, 17], however, the extent of the corresponding risk difference associated with CS is greater in Brazil than in those countries, because of its higher MMR. Another important aspect to consider is the role of over-medicalization as a threat to quality and improved health outcomes, which emerges in stage IV of the obstetric transition (MMR < 50/100 000 births) [36] and that high CS rates may be an obstacle for further reducing MMR.

### Conclusion

Although the perceived safety of CS has increased its acceptance for both clinicians and patients [5], our study suggests that the procedure is independently associated with increased risk of postpartum maternal death. Clinicians and patients should consider this fact when balancing the benefits and risks of this procedure. Governmental policies directed to women's health need to account that a reduction in excessive CS rates may prevent maternal deaths.

## Supporting Information

S1 Fig Selection of controls. (DOCX)

S1 Table Factors associated with caesarean section among controls. (DOC)

S2 Table Cause-specific postpartum maternal mortality associated with caesarean delivery in term deliveries only. Adjusted for region, type of hospital, age, schooling, parity and previous caesarean section.
(DOC)

S3 Table Sensitivity analysis: comparison of crude and adjusted OR's excluding and not excluding maternal near misses and women with obstetric conditions from the controls. Adjusted for region, type of hospital, age, schooling, parity, premature birth and previous caesarean section.
(DOC)

## References

[1] Vogel JP, Betran AP, Vindevoghel N, Souza JP, Torloni MR, Zhang J, et al Use of the Robson classification to assess caesarean section trends in 21 countries: a secondary analysis of two WHO multicountry surveys. Lancet Glob Health. 2015;3(5):e260–70. 10.1016/S2214-109X(15)70094-X 25866355

[2] Macfarlane A, Blondel B, Mohangoo A, Cuttini M, Nijhuis J, Novak Z, et al Wide differences in mode of delivery within Europe: risk-stratified analyses of aggregated routine data from the Euro-Peristat study. BJOG. 2015. 10.1111/1471-0528.13284 25753683

[3] Brasil. Ministério da saúde. Nascimentos por residência da mãe por ano tipo de parto / Live births by mother's residence by year and type of delivery Brasília: Ministério da Saúde; 2014 [cited 2015 21 April]. Available from: http://tabnet.datasus.gov.br/cgi/tabcgi.exe?sinasc/cnv/nvuf.def.

[4] Nakamura-Pereira M, Leal MC, Esteves-Pereira AP, Domingues RMSM, Torres JA, Dias MAB, et al Use of Robson classification to assess cesarean section rate in Brazil: the role of source of payment for childbirth. IN PRESS. Reprod Health. 2016. 10.1186/s12978-016-0228-7 PMC5073850 27766941

[5] Ramires de Jesus G, Ramires de Jesus N, Peixoto-Filho FM, Lobato G. Caesarean rates in Brazil: what is involved? BJOG. 2015;122(5):606–9. 10.1111/1471-0528.13119 25327984

[6] Domingues RMSM, Dias MAB, Nakamura-Pereira M, Torres JA, d'Orsi E, Pereira APE, et al Process of decision-making regarding the mode of birth in Brazil: from the initial preference of women to the final mode of birth. Cad Saude Publica. 2014;30:S101–S16. 10.1590/0102-311x00105113 25167169

[7] Villar J, Valladares E, Wojdyla D, Zavaleta N, Carroli G, Velazco A, et al Caesarean delivery rates and pregnancy outcomes: the 2005 WHO global survey on maternal and perinatal health in Latin America. Lancet. 2006;367(9525):1819–29. 16753484 10.1016/S0140-6736(06)68704-7

[8] Chongsuvivatwong V, Bachtiar H, Chowdhury ME, Fernando S, Suwanrath C, Kor-Anantakul O, et al Maternal and fetal mortality and complications associated with cesarean section deliveries in teaching hospitals in Asia. J Obstet Gynaecol Res. 2010;36(1):45–51. 10.1111/j.1447-0756.2009.01100.x 20178526

[9] Liu S, Liston RM, Joseph KS, Heaman M, Sauve R, Kramer MS. Maternal mortality and severe morbidity associated with low-risk planned cesarean delivery versus planned vaginal delivery at term. Cmaj. 2007;176(4):455–60. 17296957 10.1503/cmaj.060870 PMC1800583

[10] Villar J, Carroli G, Zavaleta N, Donner A, Wojdyla D, Faundes A, et al Maternal and neonatal individual risks and benefits associated with caesarean delivery: multicentre prospective study. Bmj. 2007;335(7628):1025 17977819 10.1136/bmj.39363.706956.55 PMC2078636

[11] Ye J, Betran AP, Guerrero Vela M, Souza JP, Zhang J. Searching for the optimal rate of medically necessary cesarean delivery. Birth. 2014;41(3):237–44. 10.1111/birt.12104 24720614

[12] Szwarcwald CL, Escalante JJC, Rabello Neto Dd L, Souza Junior PRBd, Victora CG. Estimation of maternal mortality rates in Brazil, 2008–2011. Cad Saude Publica. 2014;30:S71–S83. 10.1590/0102-311x00125313 25167192

[13] Morse ML, Fonseca SC, Barbosa MD, Calil MB, Eyer FP. [Maternal mortality in Brazil: what has the scientific literature shown in the last 30 years?]. Cad Saude Publica. 2011;27(4):623–38. 21603746 10.1590/s0102-311x2011000400002

[14] Vadnais M, Sachs B. Maternal mortality with cesarean delivery: a literature review. Semin Perinatol. 2006;30(5):242–6. 17011393 10.1053/j.semperi.2006.07.014

[15] Fawole AO, Shah A, Fabanwo AO, Adegbola O, Adewunmi AA, Eniayewun AB, et al Predictors of maternal mortality in institutional deliveries in Nigeria. Afr Health Sci. 2012;12(1):32–40. 23066417 PMC3462508

[16] Kamilya G, Seal SL, Mukherji J, Bhattacharyya SK, Hazra A. Maternal mortality and cesarean delivery: an analytical observational study. J Obstet Gynaecol Res. 2010;36(2):248–53. 10.1111/j.1447-0756.2009.01125.x 20492373

[17] Deneux-Tharaux C, Carmona E, Bouvier-Colle MH, Breart G. Postpartum maternal mortality and cesarean delivery. Obstet Gynecol. 2006;108(3 Pt 1):541–8. 16946213 10.1097/01.AOG.0000233154.62729.24

[18] Lilford RJ, van Coeverden de Groot HA, Moore PJ, Bingham P. The relative risks of caesarean section (intrapartum and elective) and vaginal delivery: a detailed analysis to exclude the effects of medical disorders and other acute pre-existing physiological disturbances. Br J Obstet Gynaecol 1990;97: 883–92. 2223678 10.1111/j.1471-0528.1990.tb02442.x

[19] Vasconcellos MTLd, Silva PLdN, Pereira APE, Schilithz AOC, Souza Junior PRBd, Szwarcwald CL. Sampling design for the Birth in Brazil: National Survey into Labor and Birth. Cad Saude Publica. 2014;30:S49–S58. 10.1590/0102-311x00176013 25167189

[20] Leal MC, Moura da Silva AA, Dias MA, Nogueira da Gama SG, Rattner D, Moreira ME, et al Birth in Brazil: national survey into labour and birth. Reprod Health. 2012;9(1):15. 22913663 10.1186/1742-4755-9-15 PMC3500713

[21] Frias PGd, Pereira PMH, Andrade CLTd, Lira PICd, Szwarcwald CL. Evaluation of data on mortality and live births in Pernambuco State, Brazil. Cad Saude Publica. 2010;26:671–81. 20512208 10.1590/s0102-311x2010000400010

[22] Brasil. Ministério da Saúde. Guia de vigilância epidemiológica do óbito materno / Guidence on maternal death surveillance. Secretaria de Vigilância em Saúde, Ministério da Saúde, Brasília—DF. 2009.

[23] Camargo KR Jr., Coeli CM. [Reclink: an application for database linkage implementing the probabilistic record linkage method]. Cad Saude Publica. 2000;16(2):439–47. 10883042 10.1590/s0102-311x2000000200014

[24] Frias PG, Szwarcwald CL, Lira PI. [Evaluation of information systems on live births and mortality in Brazil in the 2000s]. Cad Saude Publica. 2014;30(10):2068–280. 25388310 10.1590/0102-311x00196113

[25] Dias MAB, Domingues RMSM, Schilithz AOCa, Nakamura-Pereira M, Diniz CSG, Brum IR, et al Incidence of maternal near miss in hospital childbirth and postpartum: data from the Birth in Brazil study. Cad Saude Publica. 2014;30:S169–S81. 10.1590/0102-311x00154213 25167176

[26] Say L, Souza JP, Pattinson RC, Mortality WHOwgoM, Morbidity c. Maternal near miss—towards a standard tool for monitoring quality of maternal health care. Best Pract Res Clin Obstet Gynaecol. 2009;23(3):287–96. 10.1016/j.bpobgyn.2009.01.007 19303368

[27] Gabriel GP, Chiquetto L, Morcillo AM, Ferreira Mdo C, Bazan IG, Daolio LD, et al [Evaluation of data on live birth certificates from the Information System on Live Births (SINASC) in Campinas, Sao Paulo, 2009]. Rev Paul Pediatr. 2014;32(3):183–8. 10.1590/0103-0582201432306 25479847 PMC4227338

[28] Lumbiganon P, Laopaiboon M, Gülmezoglu AM, Souza JP, Taneepanichskul S, Ruyan P, at al. Method of delivery and pregnancy outcomes in Asia: the WHO global survey on maternal and perinatal health 2007–08. Lancet. 2010 2 6;375(9713):490–9. 10.1016/S0140-6736(09)61870-5 20071021

[29] Sheldon WR, Blum J, Vogel JP, Souza JP, Gulmezoglu AM, Winikoff B, et al Postpartum haemorrhage management, risks, and maternal outcomes: findings from the World Health Organization Multicountry Survey on Maternal and Newborn Health. BJOG. 2014;121 Suppl 1:5–13. 10.1111/1471-0528.12636 24641530

[30] Rocha Filho EA, Costa ML, Cecatti JG, Parpinelli MA, Haddad SM, Pacagnella RC, et al Severe maternal morbidity and near miss due to postpartum hemorrhage in a national multicenter surveillance study. Int J Gynaecol Obstet. 2015;128(2):131–6. 10.1016/j.ijgo.2014.08.023 25468058

[31] Azevedo Bittencourt SD, Costa Reis LG, Ramos MM, Rattner D, Rodrigues PL, Neves DC, et al Structure in Brazilian maternity hospitals: key characteristics for quality of obstetric and neonatal care. Cad Saude Publica. 2014;30 Suppl 1:S1–12. 10.1590/0102-311x00176913 25167180

[32] Schantz-Dunn J, N M. The use of blood in obstetrics and gynecology in the developing world. Rev Obstet Gynecol. 2011;4(2):86–91. 22102932 PMC3218550

[33] Ophir E, Strulov A, Solt I, Michlin R, Buryanov I, Bornstein J. Delivery mode and maternal rehospitalization. Arch Gynecol Obstet. 2008;277(5):401–4. 17922286 10.1007/s00404-007-0476-4

[34] Pacagnella RC, Cecatti JG, Parpinelli MA, Sousa MH, Haddad SM, Costa ML, et al Delays in receiving obstetric care and poor maternal outcomes: results from a national multicentre cross-sectional study. BMC Pregnancy Childbirth. 2014;14:159 10.1186/1471-2393-14-159 24886330 PMC4016777

[35] Pfitscher LC, Cecatti JG, Haddad SM, Parpinelli MA, Souza JP, Quintana SM, et al The role of infection and sepsis in the Brazilian Network for Surveillance of Severe Maternal Morbidity. Trop Med Int Health. 2016;21(2):183–93. 10.1111/tmi.12633 26578103

[36] Souza JP, Tuncalp O, Vogel JP, Bohren M, Widmer M, Oladapo OT, et al Obstetric transition: the pathway towards ending preventable maternal deaths. BJOG. 2014;121 Suppl 1:1–4. 10.1111/1471-0528.12735 24641529

