# Supplementary material for: Caesarean Delivery and Postpartum Maternal Mortality: A Population-Based Case Control Study in Brazil
Source: PLoS One. 2016 Apr 13;11(4):e0153396. doi: 10.1371/journal.pone.0153396 (PMC4830588; doi:10.1371/journal.pone.0153396)
Supplement: S1 Fig — (DOCX) [file pone.0153396.s001.docx]

Post-partum women after singleton birth

who were alive 42 days after birth and

not classified as maternal nearmiss

n= 9,221

Exclusion:

Maternal deaths: n= 2

Maternal nearmiss: n= 103

Exclusion:

Multiple births: n= 88

Post-partum women after singleton birth:

n= 9,326

Post-partum women who delivered in one of the public or mixed hospitals sampled for Birth in Brazil Study, in the eight states, 2011:

n= 9,414

**Selection of Controls**
