# Supplementary material for: Caesarean Delivery and Postpartum Maternal Mortality: A Population-Based Case Control Study in Brazil
Source: PLoS One. 2016 Apr 13;11(4):e0153396. doi: 10.1371/journal.pone.0153396 (PMC4830588; doi:10.1371/journal.pone.0153396)
Supplement: S1 Table — (DOC) [file pone.0153396.s002.doc]

| Table S1 - Factors associated with cesarean section among controls | | | | | | |
| --- | --- | --- | --- | --- | --- | --- |
|  |  | Vaginal | | Cesarean | | P-value |
|  |  | n | % | n | % |  |
| Region | Southeast (ref.) | 3337 | 54.2 | 2815 | 45.8 | <0.001 |
|  | South | 1301 | 52.3 | 1188 | 47.7 |  |
|  | Midwest | 257 | 44.3 | 323 | 55.7 |  |
|  |  |  |  |  |  |  |
| Type of hospital | Public (ref.) | 2446 | 63.3 | 1417 | 36.7 | <0.001 |
|  | Mixed | 2450 | 45.7 | 2909 | 54.3 |  |
|  |  |  |  |  |  |  |
| Age in years | 10-19 | 1146 | 65.1 | 614 | 34.9 | <0.001 |
|  | 20-24 (ref.) | 1665 | 59.2 | 1149 | 40.8 |  |
|  | 25-29 | 1064 | 48.5 | 1132 | 51.5 |  |
|  | 30-34 | 644 | 42.0 | 890 | 58.0 |  |
|  | ≥ 35 | 374 | 40.9 | 541 | 59.1 |  |
|  |  |  |  |  |  |  |
| Years of education | ≤ 3 | 197 | 69.1 | 88 | 30.9 | <0.001 |
|  | 4-7 | 1131 | 60.5 | 739 | 39.5 |  |
|  | 8-11 | 3017 | 53.4 | 2637 | 46.6 |  |
|  | ≥ 12 (ref.) | 543 | 39.1 | 845 | 60.9 |  |
|  |  |  |  |  |  |  |
| Skin color | Non white | 2883 | 56.7 | 2202 | 43.3 | <0.001 |
|  | White (ref.) | 2009 | 48.6 | 2123 | 51.4 |  |
|  |  |  |  |  |  |  |
| Number of previous births | 0 | 2060 | 49.9 | 2068 | 50.1 | <0.001 |
|  | 1 to 2 (ref.) | 2233 | 54.0 | 1903 | 46.0 |  |
|  | ≥ 3 | 603 | 63.0 | 354 | 37.0 |  |
|  |  |  |  |  |  |  |
| Previous c-section | 0 (ref.) | 2408 | 81.1 | 561 | 18.9 | <0.001 |
|  | 1 | 394 | 25.0 | 1180 | 75.0 |  |
|  | ≥ 2 | 18 | 3.5 | 503 | 96.5 |  |
|  |  |  |  |  |  |  |
|  | Primiparous | 2059 | 49.9 | 2068 | 50.1 |  |
| Prematurity | No (ref.) | 4374 | 53.4 | 3812 | 46.6 | 0.22 |
|  | Yes | 473 | 51.3 | 449 | 48.7 |  |
