# Supplementary material for: Caesarean Delivery and Postpartum Maternal Mortality: A Population-Based Case Control Study in Brazil
Source: PLoS One. 2016 Apr 13;11(4):e0153396. doi: 10.1371/journal.pone.0153396 (PMC4830588; doi:10.1371/journal.pone.0153396)
Supplement: S2 Table — Adjusted for region, type of hospital, age, schooling, parity and previous caesarean section. (DOC) [file pone.0153396.s003.doc]

| Table S2 - Cause-specific postpartum maternal mortality associated with cesarean delivery in term deliveries only | | | | | | | | | | | | | | |
| --- | --- | --- | --- | --- | --- | --- | --- | --- | --- | --- | --- | --- | --- | --- |
|  | Cases (52) | | | |  | Controls (8 185) | | | |  | Crude | | Adjusted * | |
|  | Vaginal | | Caesarean | |  | Vaginal | | Caesarean | |  |
|  | n | % | n | % |  | n | % | n | % |  | OR | 95% CI | OR adj. | 95% CI |
| All causes | 13 | 25.0 | 39 | 75.0 |  | 4 374 | 53.4 | 3 811 | 46.6 |  | 3.4 | (1.8 - 6.5) | 5.0 | (2.2 - 10.3) |
| Postpartum haemorrhage | 4 | 14.3 | 24 | 85.7 |  |  |  |  |  |  | 6.9 | (2.4 - 19.9) | 10.3 | (3.2 - 32.8) |
| Puerperal infection | 7 | 53.8 | 6 | 46.2 |  |  |  |  |  |  | 0.9 | (0.33 - 2.9) | 1.3 | (0.4 - 4.6) |
| Thomboembolism and other causes combined^§^ | 2 | 18.2 | 9 | 81.8 |  |  |  |  |  |  | 5.1 | (1.1 - 23.9) | 8.1 | (1.5 - 45.2) |
| * Adjusted for region, type of hospital, age, schooling, parity and previous c-section. | | | | | | | | | | | | |  |  |
| § Includes amniotic fluid embolism, complications of anesthesia and unspecified obstetric death after hospital discharge. | | | | | | | | | | | | | |  |
