# Supplementary material for: Caesarean Delivery and Postpartum Maternal Mortality: A Population-Based Case Control Study in Brazil
Source: PLoS One. 2016 Apr 13;11(4):e0153396. doi: 10.1371/journal.pone.0153396 (PMC4830588; doi:10.1371/journal.pone.0153396)
Supplement: S3 Table — Adjusted for region, type of hospital, age, schooling, parity, premature birth and previous caesarean section. (DOC) [file pone.0153396.s004.doc]

| Table S3 - Sensitivity analysis: comparison of crude and adjusted OR's excluding and not excluding maternal near misses and women with obstetric conditions from the controls | | | | | | | |
| --- | --- | --- | --- | --- | --- | --- | --- |
|  |  | n | % | Crude | | Adjusted* | |
|  |  |  |  | OR | 95% IC | OR adj. | 95% IC |
| **Cases** | All | 73 | 100.0 | - | - | - | - |
|  | Vaginal | 26 | 35.6 | - | - | - | - |
|  | Caesarean | 47 | 64.4 | - | - | - | - |
| **Controls^1^** | All | 9 176 | 100.0 | - | - | - | - |
|  | Vaginal | 4 846 | 52.8 | - | - | - | - |
|  | Caesarean | 4 330 | 47.2 | 2.02 | (1.26 - 3.29) | 2.81 | (1.58 - 4.97) |
| **Controls^2^** | All | 9 073 | 100.0 | - | - | - | - |
|  | Vaginal | 4 817 | 53.1 | - | - | - | - |
|  | Caesarean | 4 256 | 46.9 | 2.04 | (1.27 - 3.31) | 2.87 | (1.63 - 5.06) |
| **Controls^3^** | All | 9 028 | 100.0 | - | - | - | - |
|  | Vaginal | 4 847 | 53.7 | - | - | - | - |
|  | Caesarean | 4 181 | 46.3 | 2.09 | (1.31 - 3.37) | 2.96 | (1.74 - 5.17) |
| * Adjusted for region, type of hospital, age, schooling, parity, premature birth and previous c-section. | | | | | | | |
| 1 All women with single pregnancy interviewed in the Birth in Brazil study that were alive at 42 days after birth. | | | | | | | |
| 2 All women with single pregnancy interviewed in the Birth in Brazil study that were alive at 42 days after birth and not classified as maternal nearmiss. | | | | | | | |
| 3 All women with single pregnancy interviewed in the Birth in Brazil study that were alive at 42 days after birth, not classified as maternal nearmiss and without obstetric conditions potentially related to emergencies before birth (which included: eclampsia, placenta praevia or accreta, abruption placenta and severe infeccion). | | | | | | | |
